# Two Sequential Clinical Isolates of *Candida glabrata* with Multidrug-Resistance to Posaconazole and Echinocandins

**DOI:** 10.3390/antibiotics10101217

**Published:** 2021-10-07

**Authors:** Qiqi Wang, Yun Li, Xuan Cai, Ruoyu Li, Bo Zheng, Ence Yang, Tianyu Liang, Xinyu Yang, Zhe Wan, Wei Liu

**Affiliations:** 1 Department of Dermatology and Venerology, Peking University First Hospital, Beijing 100034, China; 1911110247@bjmu.edu.cn (Q.W.); mycolab@126.com (R.L.); ericliang0123@163.com (T.L.); yanxinyu1994@bjmu.edu.cn (X.Y.); zym@tsinghua.edu.cn (Z.W.); 2 National Clinical Research Center for Skin and Immune Diseases, Beijing 100034, China; 3 Research Center for Medical Mycology, Peking University, Beijing 100034, China; 4 Beijing Key Laboratory of Molecular Diagnosis on Dermatoses, Beijing 100034, China; 5 Institute of Clinical Pharmacology, Peking University First Hospital, Beijing 100034, China; liyun19702@sina.com (Y.L.); doctorzhengbo@163.com (B.Z.); 6 Department of Clinical Laboratory, Renmin Hospital of Wuhan University, Wuhan 430060, China; caixuanyx@hotmail.com; 7 Department of Microbiology & Infectious Disease Center, School of Basic Medical Sciences, Peking University Health Science Center, Beijing 100191, China; yangence@pku.edu.cn

**Keywords:** *Candida glabrata*, multidrug-resistance, echinocandins, triazoles, posaconazole, FK520

## Abstract

*Candida glabrata* is one of the most prevalent causative pathogens of invasive candidiasis, and multidrug-resistant strains are emerging. We identified two clinical isolates of *C. glabrata*, BMU10720 and BMU10722 sequentially isolated from one patient with multidrug-resistance to posaconazole (POS), caspofungin (CAS), micafungin (MCF), and anidulafungin (ANF). Overexpression of *ERG11* in BMU10720 and *CDR1* in BMU10722 were detected at basal level. When exposed to POS, *CDR1* was significantly up-regulated in both isolates compared with susceptible reference strain, while *ERG11* was up-regulated considerably only in BMU10720. *PDR1* sequencing revealed that both isolates harbored P76S, P143T, and D243N substitutions, while *ERG11* was intact. Cdr1 inhibitor FK520 reversed POS-resistance by down-regulating *ERG11* expression. *FKS* sequencing revealed that both isolates harbored S663P substitution in *FKS2*, and four single nucleotide polymorphisms (SNPs) existed in *FKS2* genes between BMU10720 and BMU10722, while *FKS1* was intact. Both *FKS1* and *FKS2* were up-regulated by CAS in BMU10720 and BMU10722. FK520 down-regulated *FKS2* expression induced by CAS through inhibiting calcineurin, resulting in synergic effect with echinocandins as well as Congo Red and Calcofluor White, two cell wall-perturbing agents. In conclusion, the multidrug-resistance of *C. glabrata* isolates in our study was conferred by different mechanisms. *CDR1* and *ERG11* overexpression in one isolate and only *CDR1* overexpression in the other isolate may mediate POS-resistance. S663P mutation in *FKS2* and up-regulation of *FKS2* may contribute to echinocandin-resistance in both isolates.

## 1. Introduction

Invasive candidiasis (IC) is a life-threatening disease with substantial morbidity and mortality, especially among immunosuppressed hosts [1]. Although *Candida albicans* remains the most prevalent *Candida* species causing IC, the past decades have witnessed an epidemiological shift to non-*albicans* spp., among which *Candida glabrata* is one of the most common pathogens and exhibits an increasing trend [1,2,3]. *C. glabrata* has become the most prevalent non-*albicans Candida* spp. for candidemia in the United States and the third most common in China [4,5]. Triazoles, such as fluconazole (FLC), itraconazole (ITC), voriconazole (VRC), and posaconazole (POS), acting by binding and inhibiting the 14-α-demethylase, a key enzyme of the ergosterol biosynthetic pathway encoded by *ERG11*, remain the most widely used antifungals. *C. glabrata* is of particular concern due to its high rate of reduced susceptibility to triazoles, with nearly 20% isolates exhibiting intrinsic resistance and susceptible isolates acquiring resistance rapidly [6]. To address this issue, echinocandins, such as caspofungin (CAS), micafungin (MCF), and anidulafungin (ANF), targeting β-1,3-glucan synthase (encoded by *FKS1* in most *Candida* spp. and *FKS1*/*FKS2* in *C. glabrata*), a key enzyme in the biosynthesis of a major structural component of the fungal cell wall β-1,3-glucan, are recommended as first-line agents for the treatment of invasive *C. glabrata* infections [7]. Troublingly, echinocandin-resistance is emerging, and on the rise, and becoming a growing threat to successful clinical management [8]. Echinocandin-resistance among *C. glabrata* isolates ranges from 3–5% in population-based studies, and some centers even report as high as 10–15% [9], but it is less than 1% in China [10,11]. Alarmingly, *C. glabrata* often presents as multidrug-resistance, with nearly one-third of echinocandin-resistant isolates also being non-susceptible to triazoles, leaving extremely few options to treat patients infected with multidrug-resistant isolates [12].

Triazole-resistance in *C. glabrata* is mainly mediated by functional mutations in the transcription regulator *PDR1*, which is responsible for the overexpression of ATP binding cassette (ABC) family multidrug transporters, such as *CDR1*, *CDR2*, and *SNQ2* [13,14]. Mutations in the *ERG11* [15] and overexpression of *ERG11* were also reported in sporadic resistant clinical isolates [16,17], and one case of *EGR11* overexpression was resulted from a chromosomal duplication [18]. Mechanisms of echinocandin-resistance appear to be more straightforward, mainly associating with mutations in hotspot (HS) regions of *FKS1* and *FKS2* genes encoding a catalytic subunit of β-1,3-glucan synthase [12]. Drug adaptation is also a key intermediate leading to echinocandin-resistance in *C. glabrata*, among which, and of particular significance, is the cell wall integrity (CWI) pathway regulating glucan synthesis through upregulation of the *FKS* genes and activation of PKC [12].

However, multidrug-resistance in *C. glabrata* was rarely reported in China, and the mechanisms underlying multidrug-resistance in this pathogen have been poorly explored and remain obscure. Here, we report two isolates of *C. glabrata* recovered from one patient at a three-day interval, which are both resistant to all the three echinocandins and non-wide-type (NWT) to POS as well. In addition, the mechanisms responsible for the multidrug-resistance phenotype were also elucidated.

## 2. Materials and Methods

### 2.1. Strains and Molecular Identification

A 53-year-old male was admitted to Renmin Hospital of Wuhan University with a complaint of low back pain accompanied by fever and chills. Abdominal computed tomography (CT) revealed renal abscess. Renal fungal abscess was speculated and empiric antifungal therapy with MCF 150 mg daily was initiated. A week later, the symptom persisted, and bedside B-ultrasound revealed deteriorated renal abscess. Ultrasound-guided percutaneous nephrostomy and drainage was performed and the drainage purulent urine culture was positive for *Candida* spp., named BMU10720. Antifungal therapy with MCF 150 mg daily was continued for another three days, but the symptom did not relive and drainage purulent urine from nephrostomy tube was cultured positive for another isolate of *Candida* spp., named BMU10722. Due to the poor response, MCF was replaced by VRC 200 mg daily. Unfortunately, the condition continued to deteriorate and nine days later he died because of sepsis and multi-organ failure.

These two isolates of *Candida* spp. BMU10720 and BMU10722 were sent to the Research Center for Medical Mycology, Peking University First Hospital, Beijing, China, for species identification and further study. Genomic DNA of BMU10720 and BMU10722 was extracted, the internal transcribed spacers (ITS) gene and large-subunit (28S) ribosomal rRNA gene D1/D2 domain sequencing was undertaken [19]. The sequences were analyzed against the CBS database (https://wi.knaw.nl/page/Pairwise_alignment, accessed on 21 May 2021), and both isolates were identified as *C. glabrata*.

### 2.2. Antifungal Susceptibility Testing

In vitro antifungal susceptibility to POS, FLC, ITC, VRC, CAS, MCF, ANF, and amphotericin B (AMB) was performed according to the Clinical and Laboratory Standards Institute (CLSI) M27-A4 document [20]. Two reference strains, *Candida parapsilosis* ATCC 22019 and *Candida krusei* ATCC 6258, were included as quality-control. The susceptible reference strain *C. glabrata* ATCC2001 was included for comparison purposes. MICs were determined after 24 h of incubation. The MIC was estimated as the lowest concentration of the antifungal, inducing at least a 50% reduction (for all antifungals except AMB) or a 100% reduction (for AMB) in fungal growth from that of the control. The MIC interpretive criteria included *C. glabrata*-specific CLSI breakpoints for FLC, CAS, MCF, and ANF [21], as well as epidemiologic cutoff values (ECVs) for VRC, ITC, POS, and AMB [22].

Antifungal susceptibility testing by E-test was performed according to the manufacturer’s instructions. Briefly, inoculum suspensions at a final concentration of 1 × 10^6^ CFU/mL were inoculated on the entire surface of each 90-mm plate containing 25 mL of RPMI 1640 medium with a sterile cotton swab. The E-test strips (Autobio, Zhengzhou, China) were placed on the center of the plate and incubated at 35 °C. The MICs were determined from the inhibition ellipse that intersected the scale on the strip after 24 h.

Antifungal susceptibility testing by disk diffusion was performed by using disks prepared in-house of POS (10 μg), FLC (80 μg), ITC (80 μg), VRC (10 μg), CAS (10 μg), MCF (10 μg), ANF (10 μg), with or without Cdr1 inhibitor FK520 (20 μg), referring to the previously described method [23].

### 2.3. Amplification and Sequence Analysis of Resistance-Related Genes

In order to detect mutations in resistance-related genes in these two multidrug-resistant *C. glabrata* isolates, the open reading frame (ORF) of the *PDR1*, *ERG11*, *FKS1*, and *FKS2* genes were amplified with the primers listed in Table 1. The amplified products were sent to the BGI Company (Beijing, China) for sequencing. The sequences were analyzed against those of reference strain (GenBank reference sequence No. *PDR1* FJ550269, *ERG11* XM_445876, *FKS1* XM_446406, and *FKS2* XM_448401) and single nucleotide polymorphisms (SNPs) were detected using Clustal Omega (https://www.ebi.ac.uk/Tools/msa/clustalo/, accessed on 15 June 2021).

### 2.4. RNA Extraction and Quantitative Real-Time Reverse-Transcription (RT)-PCR

Suspensions of *C. glabrata* isolates BMU10720, BMU10722, and ATCC2001 cells (OD_600_, 0.1) freshly prepared in YPD medium were grown at 30 °C to reach an OD_600_ of 0.5. Subsequently, each isolate was divided into 6 groups: drug-free, FK520 (added into YPD medium at a final concentration of 100 mg/L), POS (2 mg/L for BMU10720 and BMU10722, 0.12 mg/L for ATCC2001), POS+FK520 (above mentioned concentration), CAS (4 mg/L for BMU10720 and BMU10722, 0.12 mg/L for ATCC2001), CAS+FK520 (above mentioned concentration); then the cultures were incubated at 30 °C for 3 h. After treatment, the cells were centrifuged at 14,000 g for 3 min and washed twice with sterile water. Total RNA was extracted using the RNeasy Mini kit (QIAGEN Science, Germanton, MD, USA) following the manufacturer’s instructions. The RNA was then treated with RNase-free DNase (Thermo Fisher Scientific, Waltham, MA, USA) according to the manufacturer’s recommendations. According to the manufacturer’s instructions provided for the ReverAid first-strand cDNA synthesis kit (Thermo Science, Waltham, MA, USA), first-strand cDNA was amplified from 1 μg total RNA by the use of oligo (dT) primers. Real-time PCR was run on an Applied Biosystems ViiA7 real-time PCR system. Each reaction mixture volume (20 μL) contained 1 × TaqPath ProAmp master mix (Applied Biosystems, Foster City, CA, USA), 500 nM each primer, 250 nM probe, 2 μL cDNA, and nuclease-free water. The cycling conditions were as follows: 1 cycle of 95 °C for 10 min, 5 cycles of 92 °C for 15 s and 58 °C for 1 min, and 40 cycles of 92 °C for 15 s and 60 °C for 1 min. The relative quantification in gene expression was determined using the 2^−ΔΔCt^ method with the expression level of the *RDN5.8* gene for normalization [24]. The primers and fluorescent probes used were listed in Table 1.

### 2.5. Testing Susceptibility to FK520 with Other Agents

Spot assay was used to determine susceptibilities of different agents plus FK520 in *C. glabrata* strains. The indicated strains were diluted to 10^6^ CFU/mL, and 1:10 serially diluted conidial suspensions (5 μL) were inoculated onto YPD plates (plus 100 mg/L FK520 or not), with 2 mg/L CAS, 1 mg/L MCF, 1 mg/L ANF, 50 mg/L Calcofluor White (CFW), and 50 mg/L Congo Red (CR). These plates were incubated at 35 °C for 24 h before observation.

### 2.6. Statistical Analysis

The experimental data were assessed by two-way ANOVA using GraphPad Prism 8. *p*-values < 0.05 were considered statistically significant. Experiments were performed at least three independent biological replicates.

### 2.7. Data Availability

The sequences of the *PDR1*, *ERG11*, *FKS1*, and *FKS2* genes of BMU10720 and BMU10722 have been submitted to the GenBank database and assigned accession numbers MW899496-MW899503.

## 3. Results

### 3.1. C. glabrata Isolates BMU10720 and BMU10722 Are Multidrug-Resistant to POS and Echinocandins

**Figure 1:**
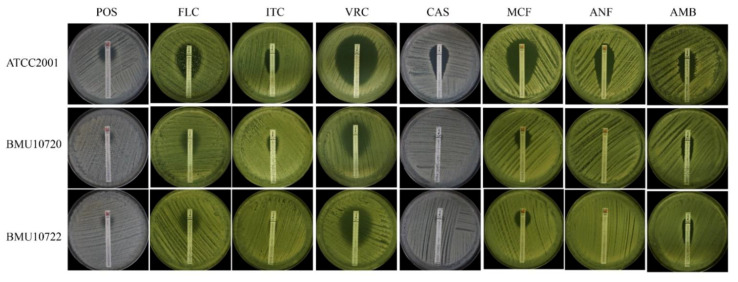
Antifungal susceptibility of ATCC2001, BMU10720, and BMU10722 performed by E-test. POS, posaconazole; FLC, fluconazole; ITC, itraconazole; VRC, voriconazole; CAS, caspofungin; MCF, micafungin; ANF, anidulafungin; AMB, amphotericin B. The inhibition zones of POS, FLC, ITC, VRC, CAS, MCF, ANF in BMU10720 and BMU10722 were smaller than those of ATCC2001, which were in accordance with the result of CLSI-M27 microdilution method. The inhibition zones of AMB in BMU10720 and BMU10722 were also smaller than that of ATCC2001, but they exhibited the same AMB MICs tested by CLSI-M27 microdilution method.

The MICs of POS against BMU10720 and BMU10722 were 4 mg/L (Table 2), which were defined as NWT to POS (ECV is 1 mg/L) [22]. Although the MICs of FLC, ITC, VRC against BMU10720 and BMU10722 (4, 2, 0.12 mg/L, respectively) were higher than those of ATCC2001, they were still defined as susceptible-dose dependent (SDD) to FLC and WT to ITC and VRC (ECVs are 4 and 0.25 mg/L, respectively) [22]. In addition, the MICs of CAS, MCF, ANF against BMU10720 and BMU10722 were 32, 8, and 4 mg/L, respectively. According to the proposed interpretative breakpoints [21], BMU10720 and BMU10722 were defined as resistant to CAS, MCF, and ANF. E-test assays showed similar results (Figure 1).

### 3.2. Four SNPs Exist in FKS2 Genes between C. glabrata Isolates BMU10720 and BMU10722

There were four SNPs in *FKS2* genes between BMU10720 and BMU10722, while the sequences of *FKS1*, *PDR1*, and *ERG11* genes were identical among them. In addition, both two isolates were identified as ST3 according to MLST analysis and also had the same genotype defined by microsatellite genotyping analysis (data not shown), indicating being closely originated with identical antifungal susceptibility profile despite being with four SNPs in *FKS2* genes.

### 3.3. Contribution of Overexpression of ERG11 and CDR1 in POS-Resistance

#### 3.3.1. Both ERG11 and CDR1 Overexpressed in BMU10720 while Only CDR1 Overexpressed in BMU10722

**Figure 2:**
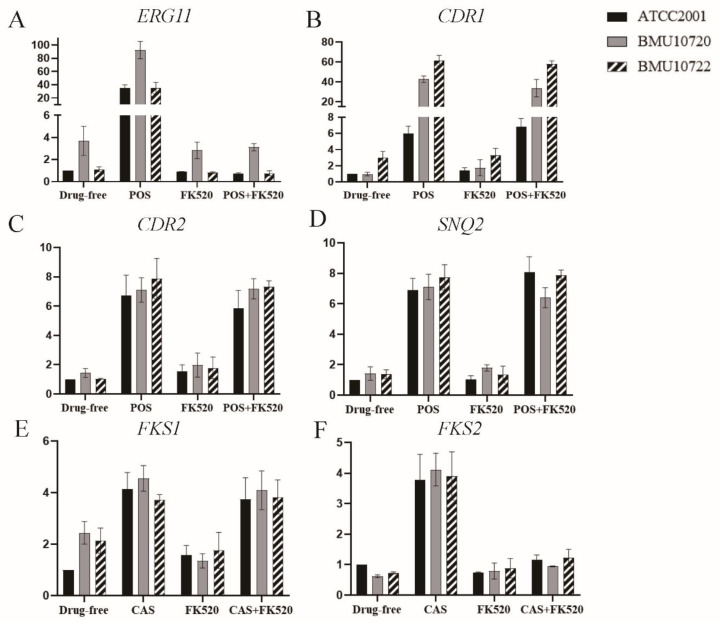
The expression level of resistance-related genes in ATCC2001, BMU10720, and BMU10722. For each target gene, the relative amount of expression was compared to that of *RDN5.8* as an internal control. The treatment of each group in detail was in the text. Data presented as means ± SE (error bars) from biological triplicates with technical triplicates. Expression data were assessed by two-way ANOVA. POS, posaconazole; CAS, caspofungin. (**A**) At basal level, the expression level of *ERG11* in BMU10720 was 4.35-fold higher than that in *C. glabrata* ATCC2001 (*p* < 0.01), while that of BMU10722 was comparable to ATCC2001 (1.12-fold vs. 1-fold, *p* > 0.99). When exposed to POS alone, the expression level of *ERG11* in BMU10720 was significantly higher than ATCC2001 (92.3-fold vs. 35.3-fold, *p* < 0.0001), while that of BMU10722 was comparable to ATCC2001 (35.1-fold vs. 35.3-fold, *p* > 0.99). FK520 alone did not change the expression level of *ERG11*. The combination of FK520 and POS sharply down-regulated *ERG11* compared to POS alone in ATCC2001, BMU10720, and BMU10722 (0.71-fold vs. 35.3-fold, *p* < 0.0001, 3.1-fold vs. 92.3-fold, *p* < 0.0001, and 0.75-fold vs. 35.1-fold, *p* < 0.0001, respectively). (**B**) At basal level, the expression level of *CDR1* in BMU10722 was 3.01-fold higher than that in *C. glabrata* ATCC2001 (*p* < 0.01), while that of BMU10720 was comparable to ATCC2001 (0.97-fold vs. 1-fold, *p* > 0.99). When exposed to POS alone, the expression level of *CDR1* in BMU10720 and BMU10722 was significantly higher than ATCC2001 (42.64-fold vs. 5.98-fold, *p* < 0.0001 and 61.31-fold vs. 5.98-fold, *p* < 0.0001, respectively). FK520 did not change the expression level of *CDR1* either in the absence or presence of POS in three isolates. (**C**) The expression level of *CDR2* was comparable in BMU10720 and BMU10722 compared to ATCC2001 at basal level (1.44-fold vs. 1-fold, *p* > 0.99 and 1.04-fold vs. 1-fold, *p* > 0.99, respectively). POS up-regulated *CDR2* in three isolates to a comparable level (7.11-fold vs. 6.73-fold, *p* > 0.99 and 7.88-fold vs. 6.73-fold, *p* = 0.85, respectively). FK520 did not change the expression level of *CDR2* either in the absence or presence of POS in three isolates. (**D**) The expression level of *SNQ2* was comparable in BMU10720 and BMU10722 compared to ATCC2001 at basal level (1.42-fold vs. 1-fold, *p* > 0.99 and 1.37-fold vs. 1-fold, *p* > 0.99, respectively). POS up-regulated *SNQ2* in three isolates to a comparable level (7.73-fold vs. 6.90-fold, *p* > 0.99 and 6.13-fold vs. 6.90-fold, *p* = 0.85, respectively). FK520 did not change the expression level of *SNQ2* either in the absence or presence of POS in three isolates. (**E**) The expression level of *FKS1* was comparable in BMU10720 and BMU10722 compared to ATCC2001 at basal level (2.44-fold vs. 1-fold, *p* = 0.11 and 2.14-fold vs. 1-fold, *p* = 0.35, respectively). CAS up-regulated *FKS1* in three isolates to a comparable level (4.56-fold vs. 4.13-fold, *p* > 0.99 and 3.72-fold vs. 4.13-fold, *p* > 0.99, respectively). FK520 did not change the expression level of *FKS1* either in the absence or presence CAS in three isolates. (**F**) The expression level of *FKS2* was comparable in BMU10720 and BMU10722 compared to ATCC2001 at basal level (0.62-fold vs. 1-fold, *p* = 0.99 and 0.73-fold vs. 1-fold, *p* > 0.99, respectively). CAS up-regulated *FKS2* in three isolates to a comparable level (4.12-fold vs. 3.78-fold, *p* > 0.99 and 3.91-fold vs. 3.78-fold, *p* > 0.99, respectively). FK520 alone did not change the expression level of *FKS2*. The combination of FK520 and CAS sharply down-regulated *FKS2* compared to CAS alone in ATCC2001, BMU10720, and BMU10722 (1.61-fold vs. 3.78-fold, *p* < 0.0001, 0.94-fold vs. 4.12-fold, *p* < 0.0001, and 1.23-fold vs. 3.91-fold, *p* < 0.0001, respectively).

Compared to *C. glabrata* ATCC2001, the expression level of *ERG11* was 4.35-fold higher in BMU10720 but was comparable in BMU10722 (Figure 2A), and the expression level of *CDR1* was 3.01-fold higher in BMU10722 but was comparable in BMU10720, when in the absence of POS (Figure 2B). When in the presence of POS, the expression level of *ERG11* and *CDR1* in BMU10720 was significantly higher than that in ATCC2001, while the expression level of *CDR2* and *SNQ2* in BMU10720 was comparable to ATCC2001. Only *CDR1* was significantly up-regulated in BMU10722 when exposed to POS than in ATCC2001, while expression of *ERG11*, *CDR2*, and *SNQ2* was comparable to ATCC2001 (Figure 2A–D).

Since disruption of the transcription factors genes *UPC2A* and *RPN4* have been identified resulting in down-regulation of *ERG11* by disturbing binding to the promoter of this gene in *C. glabrata* [25,26], the genes of these two transcriptional factors were sequenced and were found being intact in both BMU10720 and BMU10722 (data not shown). In addition, because functional alterations, except the substitutions P76S, P143T, and D243N that being considered to be MLST genotype-specific (high prevalence of ST3) [27], resulting from the mutations in the transcription regulator gene *PDR1* contribute to the overexpression of multidrug transporters genes (*CDR1*, *CDR2*, *SNQ2*) [28], *PDR1* were sequenced and substitutions P76S, P143T, and D243N were detected in both BMU10720 and BMU10722. These demonstrated that *UPC2A* and *RPN4* were not responsible for overexpression in BMU10720 and *PDR1* was not responsible for *CDR1* overexpression in BMU10720 and BMU10722.

#### 3.3.2. ERG11 Expression Level Can Be Down-Regulated by Cdr1 Inhibitor FK520 So as to Reverse POS-Resistance in BMU10720 and BMU10722

**Figure 3:**
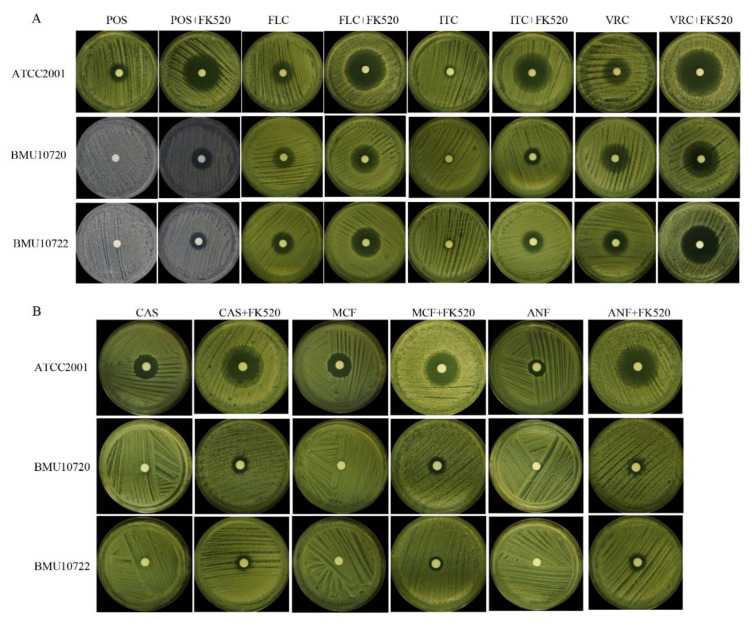
Synergic activity of FK520 with triazoles, echinocandins, and cell wall-perturbing agents. POS, posaconazole; FLC, fluconazole; ITC, itraconazole; VRC, voriconazole; CAS, caspofungin; MCF, micafungin; ANF, anidulafungin. (**A**) Disk diffusion of FK520 with triazoles: POS (10 μg), FLC (80 μg), ITC (80 μg), VRC (10 μg), without or with FK520 (20 μg). The inhibition zones of POS, FLC, ITC, and VRC combined with FK520 in ATCC2001, BMU10720, and BMU10722 were significantly larger than those of triazoles used alone. (**B**) Disk diffusion of FK520 with echinocandins: CAS (10 μg), MCF (10 μg), ANF (10 μg), without or with FK520 (20 μg). The inhibition zones of CAS, MCF, and ANF combined with FK520 in ATCC2001, BMU10720, and BMU10722 were significantly larger than those of echinocandins used alone.

Both BMU10720 and BMU10722 were resistant to POS with MICs of 4 mg/L. When being combined with Cdr1 inhibitor FK520, these two isolates showed increased susceptibility to POS with MIC of 0.12 mg/L. Besides, this synergistic effect of FK520 was also observed when being combined with FLC, ITC, and VRC (Table 1, Figure 3A). Carbonyl cyanide 3-chlorophenylhydrazone (CCCP), another efflux pump inhibitor specifically inhibiting major facilitator superfamily (MFS) transporter superfamily, was also used for comparison. And CCCP did not alter susceptibility to POS and other triazoles in *C. glabrata* isolates tested (data not shown), indicating no correlation of MFS transporter with triazole-resistance in *C. glabrata*.

Since FK520 also reversed POS-resistance in BMU10720 whose *ERG11* was overexpressed, and its structural analogue FK506 reported to enhance the susceptibility of FLC against resistant *C. glabrata* by suppressing the expression of *ERG11* and *SNQ2* [29], the expression level of *ERG11* was significantly up-regulated by exposure to POS, while no significant alteration occurred when being exposed to FK520 alone, and was sharply down-regulated to a comparable level when being exposed to the combination of POS and FK520 in *C. glabrata* ATCC2001 as well as in BMU10720 and BMU10722 (Figure 2A). The transporter genes *CDR1*, *CDR2*, and *SNQ2* were also up-regulated by exposure to POS, while the expression level of *CDR1* but not *CDR2* and *SNQ2* was slightly up-regulated when being exposed to FK520 alone (no statistical significance) in these three isolates (Figure 2B–D). When being exposed to the combination of POS and FK520, no significant alteration of the expression level in *CDR1*, *CDR2*, and *SNQ2* was observed (Figure 2B–D).

Taken together, *ERG11* and *CDR1* overexpression in BMU10720 and *CDR1* overexpression in BMU10722 may mediate POS-resistance, which could be reversed by FK520 via down-regulating *EGR11* the expression level and Cdr1 inhibition.

### 3.4. Contribution of Mutation and Up-Regulation of FKS1 and FKS2 in Echinocandin-Resistance

#### 3.4.1. BMU10720 and BMU10722 Harbor S663P Substitution in FKS2 as Well as Up-Regulation of FKS1 and FKS2 Induced by CAS

The T1987C mutation, resulting in the S663P amino acid substitution in the Hotspot 1 (HS1) region of *FKS2,* which has proven to confer echinocandin-resistance in *C. glabrata* [30], was detected in BMU10720 and BMU10722, while *FKS1* was intact in these two isolates.

Since upregulation of the *FKS* genes, which regulates glucan synthesis, is also a key intermediate leading to echinocandin-resistance in *C. glabrata* [12], the expression level of *FKS1* and *FKS2* in the absence and presence of CAS was measured. The result showed that the basal expression level of *FKS1* was higher in BMU10720 and BMU10722 compared to that in ATCC2001 (no statistical significance) (Figure 2E), and that of *FKS2* was comparable in these three isolates (Figure 2F). Being exposed to CAS, all the three isolates showed that *FKS1* and *FKS2* were up-regulated to a comparable level (Figure 2E,F).

#### 3.4.2. FKS2 Expression Level Can Be Down-Regulated by FK520 So as to Partially Reverse Echinocandin-Resistance in BMU10720 and BMU10722

Despite no significant impact on the expression level of *FKS1* and *FKS2* in these three isolates, FK520 exerted a synergistic effect with echinocandins (Table 1, Figure 3B). In addition, the expression level of *FKS2,* instead of *FKS1,* was sharply down-regulated in these three isolates when being exposed to the combination of FK520 with CAS, compared to being exposed to CAS alone (Figure 2E,F). Since FK520 and its structural analogue FK506 both inhibit calcineurin activity by binding FKBP12 and forming complexes [31] and FK506 has demonstrated the potential of down-regulating *FKS2* expression via inhibiting calcineurin so as to partially reverse *FKS2*-mediated echinocandin-resistance [32], we assumed that FK520 also down-regulated *FKS2* expression via calcineurin inhibition. This inhibitory effect of calcineurin by FK520 was further confirmed by the increased susceptibility to CFW and CR when being combined with FK520, as tested by spot assay (data not shown), since calcineurin is a key factor mediating cell wall stress-response in *C. glabrata*.

Our data are consistent with the hypothesis that echinocandin-resistance in BMU10720 and BMU10722 is mediated by S663P substitution in *FKS2*, and up-regulation of *FKS2* induced by CAS, is also a key intermediate, while the role of *FKS1* up-regulation needs to be further verified.

## 4. Discussion

In this study, we reported two isolates of *C. glabrata* BMU10720 and BMU10722 from the nephrostomy drainage purulent urine of the renal fungal abscess patient after MCF treatment for seven days and ten days, respectively. They exhibited the same antifungal susceptibility profile defined as multidrug-resistant between POS and echinocandins. Both MCF and VRC, which are not present in urine, failed to treat the patient infected with multidrug-resistant *C. glabrata*. The difference with four SNPs in *FKS2* genes between these two isolates indicates that they are two distinct isolates, despite their same source and being closely originated, revealed by MLST and microsatellite genotyping analysis.

Unlike other *Candida* spp., mutations and overexpression of *ERG11* seem to play a less critical role in triazole-resistance in *C. glabrata* [6]. Currently, only anecdotal cases of overexpression of *ERG11* in *C. glabrata* have been reported [17,18], and one was later found due to duplication of the entire chromosome containing the *ERG11* gene [18]. Although overexpression of *ERG11* plays a subtle role in triazole-resistance in *C. glabrata*, another study showed that *ERG11* up-regulation was easily inducible in susceptible *C. albicans* growing in subinhibitory concentrations of FLC [33]. In serial cultured *C. glabrata* isolates from a patient, the expression of *ERG11* was up-regulated and then decreased, with the same MIC to FLC [17]. In our study, *ERG11* was intact in both isolates while being overexpressed in BMU10720 except BMU10722. Besides, FK520 restrained *ERG11* overexpression induced by POS, so as to play a role in reversing POS-resistance in BMU10720 and BMU10722, further demonstrating the contribution of *ERG11* overexpression to POS-resistance. It has been proven that disruption of *UPC2A* and *RPN4*, the key regulators of *ERG11* expression in *C. glabrata*, had great impact on the regulation of *ERG11* [25,26]. However, no functional mutations were detected in *UPC2A* and *RPN4* of these two isolates in our study, indicating other mechanisms regulating *ERG11* expression that need to be further elucidated.

Several studies have demonstrated that overexpression of ABC transporter (mainly *CDR1*, *CDR2*, *SNQ2*) is a pivotal triazole-resistance mechanism in *C. glabrata* clinical isolates [6,34], but the MFS transporters *FLR1* and *QDR2* are not found to confer triazole-resistance [29]. Our study revealed *CDR1* overexpression in BMU10722 at the basal level while in both BMU10720 and BMU10722 under the exposure of POS. Cdr1 inhibitor FK520 restored their susceptibility to POS, further indicating the significant contribution of Cdr1 to POS-resistance. However, the *CDR1* expression was slightly up-regulated by FK520, maybe due to FK520 directly interacting with Cdr1 protein [35,36], leading to a compensatory increase in the expression level. We also used another efflux pump inhibitor CCCP for comparison, which was proven to inhibit Mdr1 belonging to MFS transporter superfamily but not Cdr1 and Cdr2 belonging to ABC transporter superfamily in *C. albicans* [37]. As expected, CCCP did not alter susceptibility to POS and other triazoles in *C. glabrata* isolates tested, confirming that MFS transporter superfamily does not confer triazole-resistance in *C. glabrata*. It has been reported that *PDR1* is comprised of four domains, namely, DNA-binding domain (DBD), inhibitory domain (ID), middle-homology domain (MHD), and activator domain (AD) [14]. Mutations in *PDR1* leading to overexpression of ABC transporter genes mainly located in ID, MDH, and AD regions [14]. In our study, P76S, P143T, and D243N in *PDR1*, located outside of these domains, was detected in both BMU10720 and BMU10722, indicating *PDR1* mutation did not contribute to POS-resistance in the two isolates. Additionally, factors other than *PDR1* regulating *CDR1* overexpression in *C. glabrata* need to be further investigated.

Intriguingly, although these two *C. glabrata* isolates were recovered from the same site of a patient at a three-day interval after MCF treatment but before VRC treatment and exhibit POS-resistance without functional mutation in *ERG11* and *PDR1*, the different expression patterns of *ERG11* and *CDR1* further indicate that they are two distinct isolates. Since the two isolates were recovered without triazole exposure, they probably harbor intrinsic POS-resistance, in line with the fact that some *C. glabrata* isolates have intrinsic resistance to triazoles [38]. On the other hand, studies also demonstrated that prior echinocandin therapy was a risk factor of both echinocandin-resistance and triazole-resistance [39], indicating that the reduced susceptibility to POS may be induced by MCF therapy in the patient. Furthermore, considering BMU10720 and BMU10722 are resistant to POS but susceptible to other triazoles, we hypothesize that the selective binding of each efflux pump with different triazoles, harboring different chemical structures, leads to various susceptibility profiles.

Unlike other *Candida* spp., *C. glabrata* exhibits a relatively high rate of echinocandin-resistance, which is mainly mediated by mutations in *FKS1* and *FKS2* [8]. In this study, the T1987C mutation resulting in the S663P substitution in the HS1 region of *FKS2*, which has been proven to be sufficient causing echinocandin-resistance in *C. glabrata* [30] and reported to be one of the most common substitutions associated with high echinocandin-MICs and therapy failure [40,41], was detected in both BMU10720 and BMU10722. In addition, the difference with four SNPs was also detected in *FKS2* between these two isolates, again confirming they are two distinct isolates. The *FKS2* mutation may be induced by MCF treatment, in concordance with a previous study that a significantly higher *FKS* mutant rate was found after MCF exposure in vivo and in vitro, among which S663P was the most frequent mutation [42]. Although *FKS* mutation is the most prevalent mechanism, drug adaptation is also a key intermediate leading to echinocandin-resistance [12]. Adaptive cellular responses make cells survive in the presence of the drug, which affords time to escape the effects of the drug by forming *FKS* mutations [8]. We further observed that CAS induced *FKS1* and *FKS2* up-regulation in echinocandin-susceptible and -resistant isolates, and up-regulation of *FKS2* but not *FKS1* was suppressed by FK520. It has been reported that inhibition of glucan synthase by echinocandins activated calcineurin-dependent stress-responses [43], and cell wall integrity (CWI) pathway activation leads to an increase in *FKS2* expression, dependent upon calcineurin [44]. Calcineurin inhibitor FK506 suppresses *FKS2* expression resulting in reversal of *FKS2*-mediated resistance in *C. glabrata* [22,45]. As an analogue of FK506, we speculated that FK520 could down-regulate *FKS2* expression also by inhibiting calcineurin activity, resulting in disturbing cell wall stress-response and sensitizing the isolates to echinocandins as well as other cell wall-perturbing agents such as CFW and CR.

Although to the best of our knowledge, this is the first study trying to elucidate the mechanisms underlying multidrug-resistance of *C. glabrata* in China, there are some limitations in this study. First, *CDR1* and *ERG11* overexpression is detected in one isolate and only *CDR1* overexpression in the other isolate was detected but cannot be explained by the mutation of the well-known transcriptional factor *PDR1*. Other cellular mechanisms may regulate the expression of *ERG11* and *CDR1* in *C. glabrata,* which require further investigations. Second, S663P mutation in *FKS2,* which has been verified sufficiently to confer echinocandin-resistance previously and induced up-regulation of *FKS1* and *FKS2* were detected in the two isolates, but the exact role of up-regulation of *FKS* needs verification.

## 5. Conclusions

Two sequential clinical isolates of *C. glabrata* with multidrug-resistance to POS and echinocandins were isolated from nephrostomy drainage purulent urine of the patient with renal abscess who failed MCF and VRC therapy. *CDR1* and *ERG11* overexpression in one isolate and only *CDR1* overexpression in the other isolate may be associated with POS-resistance. S663P mutation in *FKS2* and up-regulation of *FKS2* may contribute to echinocandin-resistance in both isolates.

## Figures and Tables

**Table 1:** Primers and fluorescent probes used in this study.

Primers	Sequences (5′-3′)	Purposes
ERG11-F	ATGTCCACTGAAAACACTTC	*ERG11* amplification and sequencing
ERG11-R	CTAGTACTTTTGTTCTGGATGTC	
PDR1-F	GGTAAAGTCATTCTTTAGCTACG	*PDR1* amplification and sequencing
PDR1-R	TACAGGCTATGCACACTGTCT	
FKS1-F	ATGTCTTACAATAATAACGGAC	*FKS1* amplification and sequencing
FKS1-W1	TTCTCCGATTTCAGCAGTTAC	*FKS1* sequencing
FKS1-W2	ACTCCAATCGAAAGAGTTCGT	
FKS1-W3	AGTTTCATCCAACTTCTAGCT	
FKS1-W4	TCAACACTGTCTTTTCCGTTG	
FKS1-W5	GATCAAGATCCTGAGAAGGAA	
FKS1-W6	TCGATGCTAACCAAGACAACT	
FKS1-W7	TGCTTTGATTTTCTACAGAGG	
FKS1-W8	CCTGGTTTCCATTTGAATAAC	
FKS1-W9	CTTCTTGGATTACAGAGACTA	
FKS1-R	TTATTTGATTGTAGACCAGGTC	*FKS1* amplification and sequencing
FKS2-F	ATGTCTTACGATCAAGGTGG	*FKS2* amplification and sequencing
FKS2-W1	CAAGGTCAAATGCCACAACAA	*FKS2* sequencing
FKS2-W2	ACAAAAAAGCAATGGAAGAGG	
FKS2-W3	TCTCCTACTTTCTACACTCAC	
FKS2-W4	GATTGCTACAGATTTCATTTTG	
FKS2-W5	TGTTAAGGATACCAAGATTCTG	
FKS2-W6	TTGATGCTAACCAAGACAACTA	
FKS2-W7	CTGGTTTCCATTTGAATAACTT	
FKS2-W8	AGATGGTTATCAAGAGGTAACA	
FKS2-W9	TTGGACTCAACCAATGAGAG	
FKS2-R	TTATTTTATAGTGGACCAGGTCTT	*FKS2* amplification and sequencing
RND5.8a	CTTGGTTCTCGCATCGATGA	real-time PCR for *RND5.8*
RND5.8b	GGCGCAATGTGCGTTCA	
RND5.8pr	6FAM-ACGCAGCGAAATGCGATACGTAATGTG-TAMRA	
CDR1a	TAGCACATCAACTACACGAACGT	real-time PCR for *CDR1*
CDR1b	AGAGTGAACATTAAGGATGCCATG	
CDR1pr	6FAM-TGCTGCTGCTTCTGCCACCTGGTT-TAMRA	
CDR2a	GTGCTTTATGAAGGCTACCAGATT	real-time PCR for *CDR2*
CDR2b	TCTTAGGACAGAAGTAACCCATCT	
CDR2pr	6FAM-TACCTTTGCGTGCTGGGCGTCACC-TAMRA	
SNQ2a	ACCATGTGTTCTGAATCAATCAAT	real-time PCR for *SNQ2*
SNQ2b	TCGACATCATTACAATACCAGAAA	
SNQ2pr	6FAM-AACTAATCGCCGCAGGTTGTGACA-TAMRA	
ERG11a	ATTGGTGTCTTGATGGGTGGTC	real-time PCR for *ERG11*
ERG11b	TCTTCTTGGACATCTGGTCTTTCA	
ERG11pr	6FAM-ACTTCCGCTGCTACCTCCGCTTGG-TAMRA	
FKS1a	TACCAACCAGAAGACCAACAGAATGG	real-time PCR for *FKS1*
FKS1b	TCACCACCGCTGATGTTTGGGT	
FKS1pr	6FAM-TGGTCAAGCCATGTACGGTGACG-TAMRA	
FKS2a	CAATTGGCAGAACACCGATCCCAA	real-time PCR for *FKS2*
FKS2b	AGTTGGGTTGTCCGTACTCATCGT	
FKS2pr	6FAM-CCAGAACAACAACAAGGTGGTGAAGGT-TAMRA	

**Table 2:** Minimal inhibitory concentrations (MICs) (mg/L) of antifungal agents alone or combined with FK520.

Isolates	POS ^1^		FLC		ITC		VRC		CAS		MCF		ANF		AMB
	−	+	−	+	−	+	−	+	−	+	−	+	−	+	−
ATCC2001	0.25	0.03	2	1	0.25	0.03	0.06	0.03	0.25	0.03	0.06	0.03	0.06	0.03	1
BMU10720	4	0.12	4	2	2	0.06	0.12	0.06	32	8	8	4	4	2	1
BMU10722	4	0.12	4	2	2	0.06	0.12	0.06	32	8	8	4	4	2	1

^1^ POS, posaconazole; FLC, fluconazole; ITC, itraconazole; VRC, voriconazole; CAS, caspofungin; MCF, micafungin; ANF, anidulafungin; AMB, amphotericin B; (−): in the absence of FK520; (+): in the presence of 100 mg/L FK520.

## Data Availability

The original data contributions presented in the study are included in the article, further inquiries can be directed to the corresponding authors.
